# Identification of IL20RB as a Novel Prognostic and Therapeutic Biomarker in Clear Cell Renal Cell Carcinoma

**DOI:** 10.1155/2022/9443407

**Published:** 2022-03-08

**Authors:** Hongda Guo, Songlin Jiang, Haoyu Sun, Benkang Shi, Yan Li, Nan Zhou, Dongqing Zhang, Hu Guo

**Affiliations:** ^1^Department of Urology, Qilu Hospital of Shandong University, 107 Wenhuaxi Road, Jinan 250012, China; ^2^Key Laboratory of Urinary Precision Diagnosis and Treatment in Universities of Shandong, Jinan, China

## Abstract

**Background:**

Clear cell renal cell carcinoma (ccRCC) is a type of life-threatening malignant tumor of the urinary system. IL20RB, interleukin 20 receptor subunit beta, is a cytokine receptor subunit coding gene and was initially found to play a vital role in human cancers, while its role in ccRCC still remains unclear.

**Methods:**

In this work, we explored the prognostic value and therapeutic potential of IL20RB in ccRCC mainly by online tools. Firstly, we used UALCAN and GEPIA to explore the expression profile and prognostic value of IL20RB in various cancers; the expression profile in tumor cell lines was also analysed with CCLE and Expression Atlas. Then, we decided to focus on ccRCC for further analysis; we further demonstrated the significant correlation between expression and clinical features by GEPIA and UALCAN. In order to reveal the potential intrinsic mechanism responsible for the upregulation of IL20RB in ccRCC, we made genetic alternation analysis and methylation analysis. cBioPortal was used for genetic alternation analysis. UALCAN, MethSurv, and Xena were used for methylation analysis. To learn details of how IL20RB might function in ccRCC, we further conducted functional analysis and immune infiltration analysis. STRING and GSEA were used to do functional analysis. TIMER was used for immune infiltration analysis; KM plotter was used for survival analysis.

**Results:**

Results show that IL20RB is upregulated in ccRCC, and low methylation may be responsible for its upregulation. Both high expression and low methylation of IL20RB predict worse survival, and both have a strong positive correlation with clinical characteristics. In addition, results indicate that there exists a crosstalk between IL20RB and neutrophils. Furthermore, the immune microenvironment could influence the prognosis predicting ability of IL20RB.

**Conclusions:**

In conclusion, IL20RB plays an important role in ccRCC and is identified as a novel prognostic and potential therapeutic biomarker in ccRCC.

## 1. Introduction

Clear cell renal cell carcinoma (ccRCC) is the predominant histological subtype and accounts for about 75% of renal cell carcinoma [1]. It is likely to recur and it threatens patient's life despite considerable efforts made in the clinical management, and its incidence is on the rise [2]. Therefore, exploring novel molecular targets is imperative as well as worthwhile, which will facilitate the innovation of more accurate diagnosis and more effective treatment. IL20RB seems a promising candidate.

Accumulating evidence has demonstrated that the immune system, a complex network of molecules, cells, tissues, and organs, plays an important role in the development of human cancers, including ccRCC [3, 4]. Recent studies focusing on cytokines and their receptors, a specific group of immune molecules, do find their potential value in prognosis and treatment of ccRCC. For instance, serum level of soluble interleukin 2 receptor was found to be able to reflect the progression of ccRCC [5]. Additionally, as is reported in a more recent study, increased serum level of soluble interleukin 2 receptor predicts worse response to interferon alpha and sequential VEGF-targeting therapy [6]. Colony-stimulating factor 1 receptor was reported to be related with unfavorable cancer-specific survival [7]. Expression of interleukin 6 receptor *α* was reported to influence response rates, in which low expression of IL6R*α* comes with a favorable response in patients treated with sunitinib [8]. In addition, CXCL13 [9], IL-8, and CXCL1 [10] were also identified as potential target in ccRCC.

IL20RB, interleukin 20 receptor subunit beta, typically forms a heterodimeric cytokine receptor with IL20RA or IL22RA1. The IL20RA/IL20RB dimer is a receptor for IL19, IL20, and IL24, and the IL22RA1/IL20RB dimer is a receptor for IL20 and IL24 [11]. IL-20RB subunit is the common chain to both receptor types. IL20RB mainly functions by binding with its ligands; these interleukins all belong to the IL20 cytokine subfamily [12, 13]. Typically, this pathway was found to be involved in immune response and tissue homeostasis. More specifically, it will facilitate the communication between epithelial cells and leukocytes. IL20 subfamily cytokines could enhance the recruitment of leukocytes, and they could also facilitate their activation at the inflammation site [14]. This pathway is identified to have the ability to trigger epidermal hyperplasia, and skin inflammation thereby is supposed to be related with the pathogenesis of chronic inflammation and autoimmune diseases [15]. Although IL20RB was initially found to be involved in many nonmalignant diseases, such as psoriasis [16], rheumatoid arthritis [17], vitiligo [18], ulcerative colitis [19], glaucoma [20], asthma [21], endometriosis [22], and chronic rhinosinusitis [23] as well as infectious diseases [24], novel discoveries indicate IL20RB could also play an important role in malignant diseases. Recently, increasing evidence supports IL20RB also plays a vital role in human cancers such as colorectal adenocarcinoma [25], breast cancer [26], and esophageal carcinoma [27], while its role in ccRCC still remains unclear.

In this study, we are aimed to make a comprehensive exploration to elucidate the potential role of IL20RB in ccRCC. Specifically, we mainly focused on the expression, prognosis value, clinical correlation, genetic alteration, methylation, function, and immune infiltration which are essential as well as valuable from a bioinformatic perspective.

## 2. Materials and Methods

### 2.1. Pan-Cancer Expression Profile of IL20RB

Expression profile of IL20RB across cancers in The Cancer Genome Atlas (TCGA) was explored in UALCAN website [28]; GEPIA [29] was used to confirm whether there truly existed statistical significance between tumor samples and normal controls. The expression profile of IL20RB in numerous tumor cell lines was obtained through the Cancer Cell Line Encyclopedia (CCLE) database [30]. Specifically, with the help of the Expression Atlas dataset, we extracted the expression profile of IL20RB in 12 different cell lines of ccRCC.

### 2.2. Expression Analysis of IL20RB in ccRCC

We verified the upregulated expression pattern of IL20RB in 3 different studies from TCGA, GEO, and Oncomine database, respectively. Paired *t*-test in TCGA-KIRC cohort was made in R software with data downloaded from TCGA database. Expression profile of IL20RB in 29 ccRCC tumor samples and 23 normal cortex samples was obtained in GDS4282 of the GEO database [31, 32]. Expression data of IL20RB in Yusenko Renal study with 26 ccRCC patients involved was obtained in Oncomine database [33]. GraphPad Prism v8.0.2 was used to generate expression boxplot of Yusenko Renal study.

### 2.3. Survival Analysis of IL20RB

We used the survival analysis tool provided in GEPIA to perform Kaplan-Meier survival estimation of overall survival (OS) and disease-free survival (DFS) in cervical squamous cell carcinoma and endocervical adenocarcinoma (CESC), esophageal carcinoma (ESCA), kidney renal clear cell carcinoma (KIRC), and lung squamous cell carcinoma (LUSC). We also downloaded the expression and clinical data from TCGA database to calculate the survival rates of 1, 3, and 5 years by R software; patients were divided into high- and low-risk groups using the median expression value. Esurv provides the optimal cutoff values for target genes in pan-cancer [34]; we used Esurv to calculate the optimal cutoff values for IL20RB in TCGA-KIRC cohort in order to provide absolute criteria for further clinical verification and practice.

### 2.4. Correlation Analysis of IL20RB Expression and Clinical Characteristics

We verified the positive correlation between IL20RB expression and ccRCC tumor stage in TCGA-KIRC cohort in UALCAN as well as GEPIA website. And the relationships of IL20RB expression with other clinical features such as patient's gender, patient's age, tumor grade, and nodal metastasis status were also evaluated by UALCAN.

### 2.5. Genetic Alteration Analysis of IL20RB in ccRCC

The cBioPortal platform [35, 36] was used to analyse the genetic alteration of IL20RB in 7 studies with 1813 samples. Survival analysis between altered group and unaltered group was also made in the survival module of the cBioPortal platform.

### 2.6. Methylation Analysis of IL20RB in ccRCC

We explored the correlation of expression and methylation of IL20RB in 400 samples from 2 studies with intact value available on the cBioPortal platform. And we made a comprehensive analysis of IL20RB methylation with clinical characteristics in UALCAN including sample type, individual cancer stage, tumor grade, patient's gender, and patient's age. In addition, we explored the role of different methylation sites in TCGA cohort of ccRCC with an online tool named MethSurv, which is a perfect platform to perform expression and survival analysis using DNA methylation data [37]. Patients were divided into high- and low-risk groups using the median methylation value. Xena platform was used for the analysis of the correlation between IL20RB expression and methylation level of different methylation sites [38].

### 2.7. Functional Analysis of IL20RB in ccRCC

We portrayed the functional protein association network of IL20RB in Homo sapiens by STRING which is an excellent online website tool to display protein-protein interaction networks [39]. And GSEA was performed to determine the different functional pathways enriched between groups classified by IL20RB expression in TCGA-KIRC cohort [40, 41].

### 2.8. Immune Infiltration Analysis

The abundances of six immune cell types (B cell, CD4 T cell, CD8 T cell, neutrophil, macrophage, and dendritic cell) in the tumor microenvironment of ccRCC were estimated using Tumor Immune Estimation Resource (TIMER) which is a web resource for evaluations of different immune cells in diverse cancer types [42, 43]. The correlation between IL20RB expression and immune cell infiltration was explored. GEPIA was applied to visualize the expression patterns of neutrophil cell markers and functional related molecules. KM plotter [44] was used to explore the different prognosis value of IL20RB in diverse immune microenvironments of ccRCC. Patients are split by auto select best cutoff value.

### 2.9. Statistical Analysis

R software (Version 4.0.2) was used to conduct Wilcoxon test for IL20RB expression analysis of paired tumor and normal samples in TCGA-KIRC cohort. GEPIA used one-way ANOVA test for expression analysis and tumor stage analysis; ∣log_2_FC | >1 and *p* value < 0.01 are set as standard of statistical significance. UALCAN used Student's *t*-test to estimate significance of difference in expression and methylation analysis. In methylation analysis made by cBioPortal and Xena, both Pearson and Spearman are used for measuring the correlation between IL20RB expression level with methylation level. In immune infiltration analysis made by TIMER, “Purity Adjustment” option was selected; the partial Spearman's correlation was used to perform this analysis. The log-rank test was used for all survival analysis. Unless otherwise specified, *p* values less than 0.05 were considered statistically significant.

## 3. Results

### 3.1. IL20RB Is Dysregulated in Tumors and Upregulated in ccRCC

**Figure 1:**
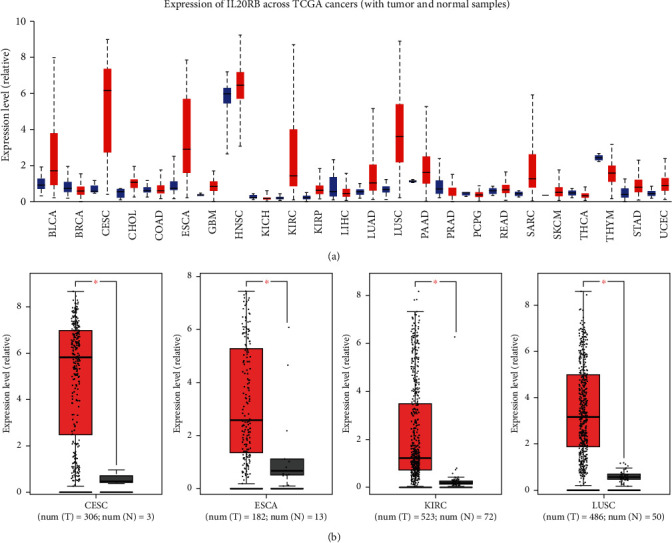
Expression of IL20RB in tumor and normal samples. (a) IL20RB expression in different types of cancer and normal tissue was investigated in UALCAN, tumor in red and normal in blue. (b) Differential significance of expression of IL20RB was suggested in TCGA-CESC, TCGA-ESCA, TCGA-KIRC, and TCGA-LUSC cohorts compared to normal tissues in the GEPIA database, tumor in red and normal in grey. Tumor abbreviations: BLCA: bladder urothelial carcinoma; BRCA: breast invasive carcinoma; CESC: cervical squamous cell carcinoma and endocervical adenocarcinoma; CHOL: cholangiocarcinoma; COAD: colon adenocarcinoma; ESCA: esophageal carcinoma; GBM: glioblastoma multiforme; HNSC: head and neck squamous cell carcinoma; KICH: kidney chromophobe; KIRC: kidney renal clear cell carcinoma; KIRP: kidney renal papillary cell carcinoma; LIHC: liver hepatocellular carcinoma; LUAD: lung adenocarcinoma; LUSC: lung squamous cell carcinoma; PAAD: pancreatic adenocarcinoma; PRAD: prostate adenocarcinoma; PCPG: pheochromocytoma and paraganglioma; READ: rectum adenocarcinoma; SARC: sarcoma; SKCM: skin cutaneous melanoma; THCA: thyroid carcinoma; THYM: thymoma; STAD: stomach adenocarcinoma; UCEC: uterine corpus endometrial carcinoma.

**Figure 2:**
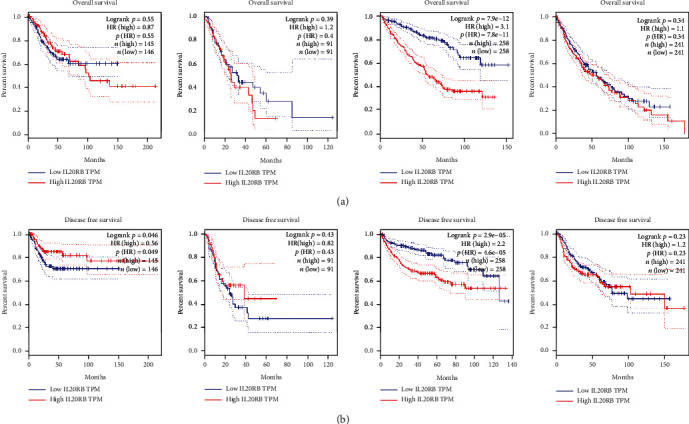
Survival analysis of IL20RB in TCGA-CESC, TCGA-ESCA, TCGA-KIRC, and TCGA-LUSC (from left to right). The relationship between IL20RB expression level with (a) overall survival and (b) disease-free survival in interested TCGA cohorts, analysed by the GEPIA database.

**Figure 3:**
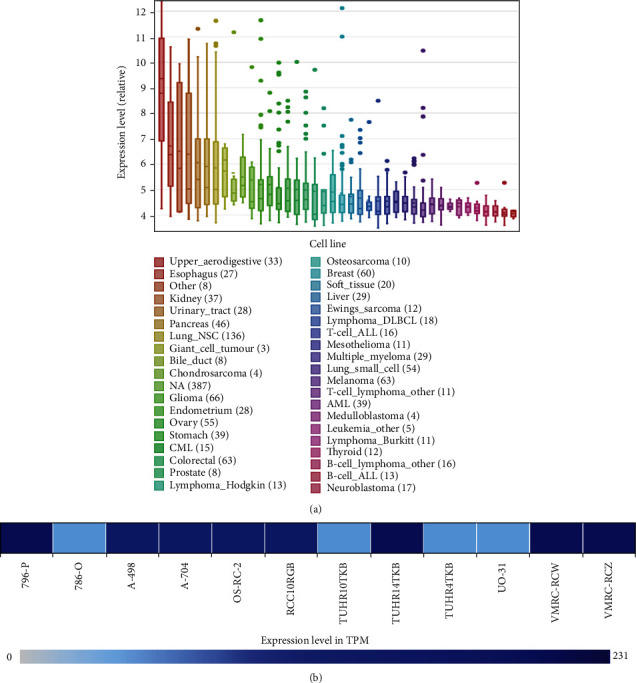
IL20RB expression in tumor cell lines. (a) IL20RB expression in a wide range of cancer cell lines was estimated by CCLE database. (b) IL20RB was relatively highly expressed in a majority of kidney cancer cell lines.

To have a perceptual panoramic view of IL20RB's role in cancers, we used the pan-cancer view function of UALCAN to visualize the expression profile of IL20RB in 24 tumor types. As is shown in Figure 1(a), IL20RB was dysregulated in a variety number of cancers, mostly upregulated. GEPIA was employed to further investigate IL20RB expression with cutoff standards of ∣log_2_FC | >1 and *p* value < 0.01; results suggested there existed differential significance of expression comparing tumor with normal samples in TCGA-CESC, TCGA-ESCA, TCGA-KIRC, and TCGA-LUSC cohorts, and IL20RB was upregulated in tumor samples in these cohorts (Figure 1(b)). Then, we explored whether or not there was prognostic significance in these cohorts, and results supported that only in TCGA-KIRC cohort there seemed to have strong correlations between IL20RB expression with both overall survival (OS) and disease-free survival (DFS) (Figure 2). At the same time, we estimated IL20RB expression in a wide range of cancer cell lines by CCLE database; results indicated that IL20RB was relatively highly expressed in kidney cancer cell lines (Figure 3(a)). Furthermore, data from Expression Atlas showed that IL20RB was relatively highly expressed in a majority of ccRCC cell lines, such as 769-P, A-498, A-704, OS-RC-2, VMRC-RCW, and VMRC-RCZ (Figure 3(b)).

**Figure 4:**
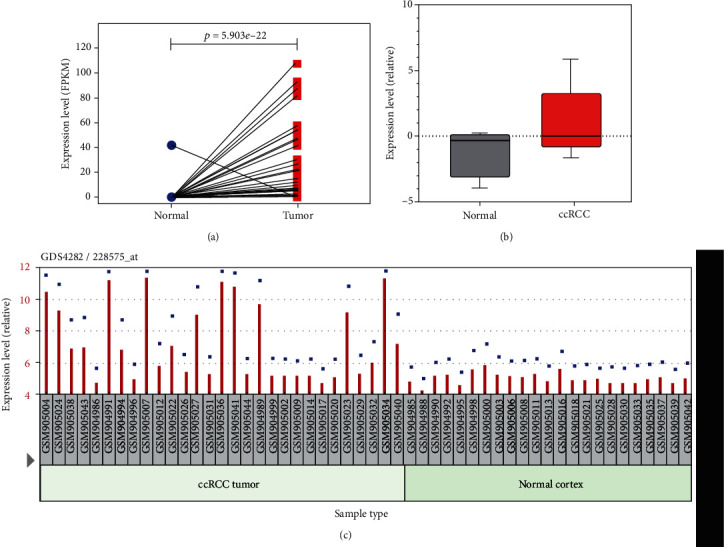
Upregulation of IL20RB in ccRCC. (a) IL20RB is upregulated in tumor tissues comparing with their normal counterparts in TCGA-KIRC cohort, tumor in red and normal in blue. (b) IL20RB is upregulated in tumor samples of Yusenko Renal study, tumor in red and normal in grey. (c) IL20RB is upregulated in GDS4282 of GEO database.

These facts urge us to make a deep exploration of IL20RB's role in ccRCC. We downloaded data of TCGA-KIRC cohort from TCGA dataset and made paired analysis; result showed that IL20RB was upregulated in tumor tissues comparing with their normal counterparts in most ccRCC patients; the *p* value is 5.903*e* − 22 (Figure 4(a)). Apart from TCGA cohort, we further investigated ccRCC studies in GEO and Oncomine database; what we discovered is that IL20RB is indeed obviously upregulated in Yusenko Renal study of Oncomine (Figure 4(b)) as well as GDS4282 of GEO (Figure 4(c)).

### 3.2. IL20RB Is Correlated with Prognosis and Clinical Characteristics in ccRCC

Survival analysis indicates high expression of IL20RB predicts a worse prognosis in ccRCC patients in terms of OS and DFS. Then, we calculated the survival rates in high and low expression groups categorized by the median expression value by R software with the data downloaded from TCGA database; the overall survival rates of 1, 3, and 5 years in the IL20RB low expression group are about 94.8%, 87.6%, and 79.5%, respectively. In the IL20RB high expression group, they dramatically dropped to only about 84.6%, 64.3%, and 46.4%. These results support IL20RB as a potential prognostic marker in ccRCC. With the help of Esurv, we calculate the optimal cutoff values for IL20RB in TCGA-KIRC cohort; result showed that the 31.4077 Transcripts Per Million (TPM) could be absolute criteria to classify patients into high- and low-risk groups based on 60-month survival rate, though further clinical verification and optimization are needed.

**Figure 5:**
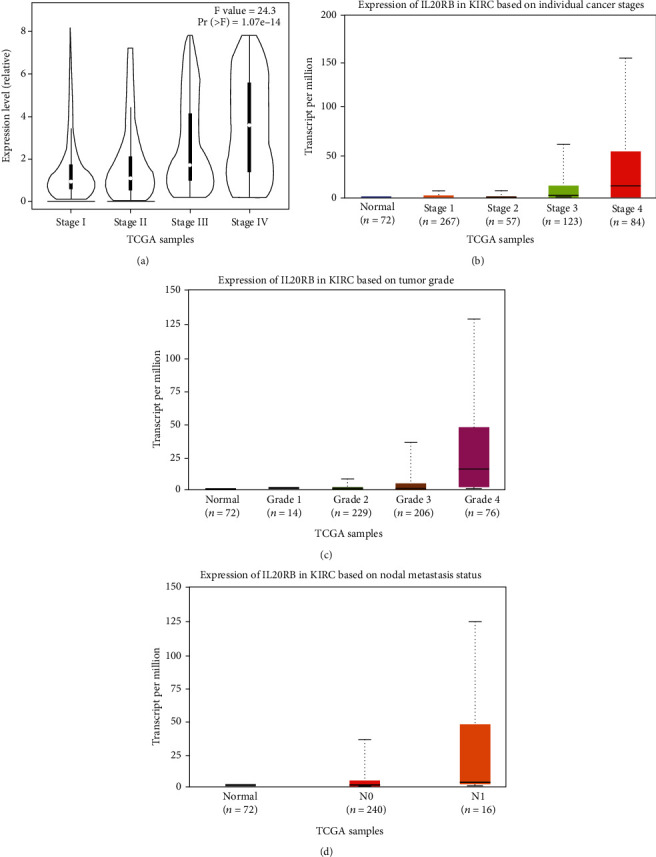
Correlation between IL20RB expression and clinical characteristics. Correlation between IL20RB expression and tumor stage analysed by (a) GEPIA and (b) UALCAN. Correlation between IL20RB expression and (c) tumor grade and (d) nodal metastasis status analysed by UALCAN.

Then, we examined IL20RB's expression with clinical characteristics in ccRCC with the assistance of GEPIA and ULCAN. As is shown in Figure 5(a), IL20RB has a strong positive correlation with tumor stage, and this result is also repeated in UALCAN. Except stage 1 vs. stage 2, stage 2 vs. stage 3, there exists statistical significance between any two groups (Figure 5(b)). A strong positive correlation was also observed between IL20RB expression and tumor grade. Results support that the higher IL20RB expression is, the higher tumor grade will be. Except normal vs. grade 1, grade 2 vs. grade 3, there exists statistical significance between any two groups. When considering about nodal metastasis status, there seems higher IL20RB expression coming with a worse nodal metastasis status; there are statistical differences in normal vs. N0 and normal vs. N1, though there was no statistical difference in N0 vs. N1 comparison (Figure 5(d)).

### 3.3. Genetic Alteration Analysis of IL20RB in ccRCC

**Figure 6:**
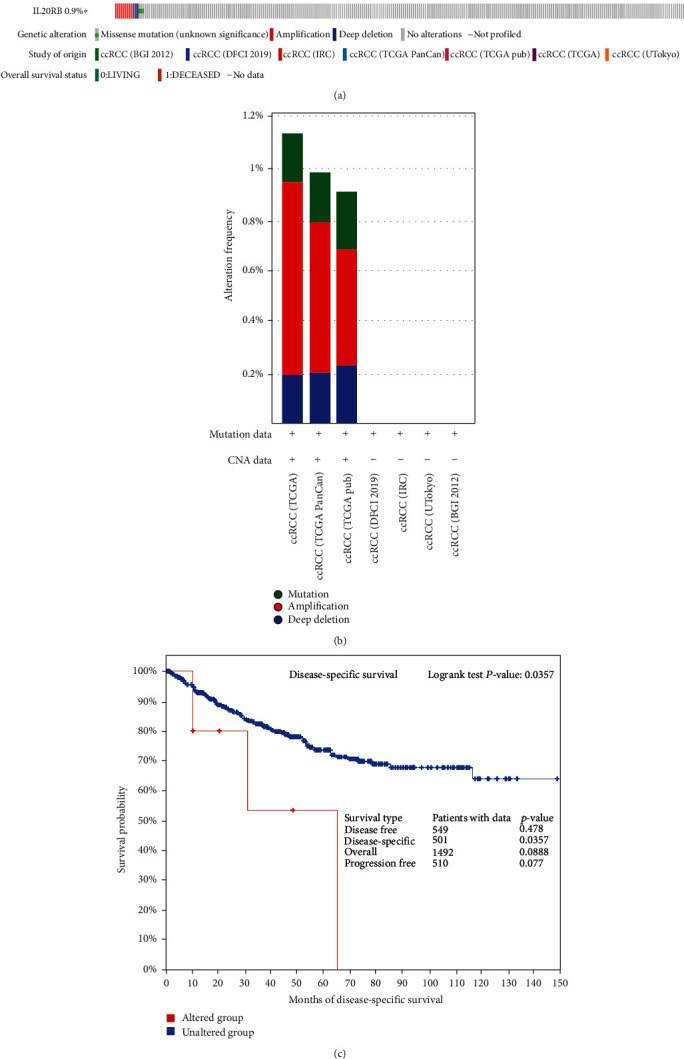
Analysis of genetic alteration of IL20RB. (a) Genetic alteration of IL20RB in 7 studies analysed by cBioPortal platform. (b) The alternation frequency of IL20RB analysed by cBioPortal platform. (c) Correlation between genetic alternation and patients' survival.

Gene alterations, including chromosomal abnormalities and genetic mutations, are thought to play an important role in the development and progression of human cancers. We decided to find out if genetic alteration of IL20RB is responsible for its high expression in ccRCC. Analysis made of 7 studies by cBioPortal platform showed the alteration rate in ccRCC is extremely low, which is only 0.9% in 1813 samples (Figure 6(a)). The most frequent alteration is amplification which may partially account for the high expression of IL20RB in a small part of patients (Figure 6(b)). Additionally, we explored the relationship between survival and genetic alternation. Results show there are no significant differences considering overall survival, disease-free survival, or progression-free survival, while there exists statistical difference between the altered group and the unaltered group when it comes to disease-specific survival; its *p* value is 0.0357 (Figure 6(c)).

### 3.4. Methylation Analysis of IL20RB in ccRCC

**Figure 7:**
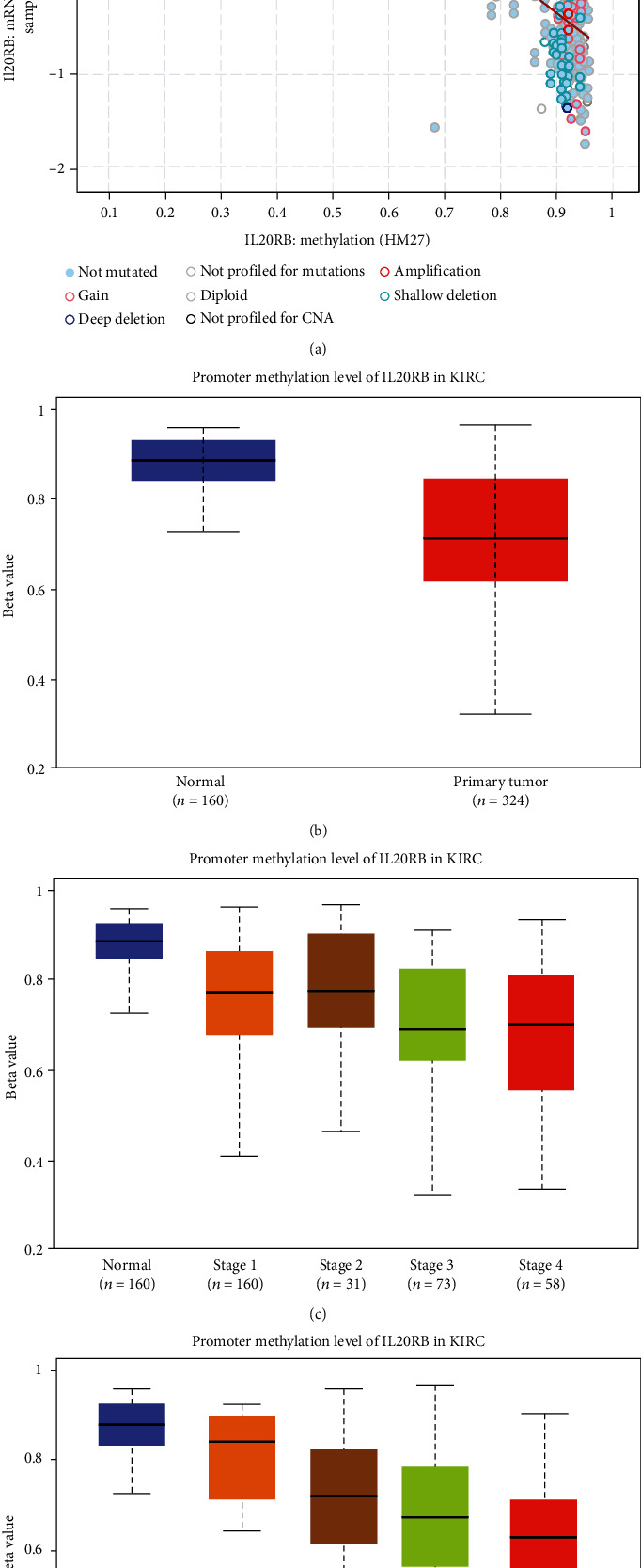
Methylation analysis of IL20RB in ccRCC patients. (a) Correlation between IL20RB expression and IL20RB methylation, analysed by cBioPortal. (b) Difference of promoter methylation level of IL20RB in tumor tissues and normal tissues. (c) Methylation shows significant variation in tumor stages. (d) Methylation shows significant variation in tumor grades.

As genetic alteration failed to be candidate explanation, we continue to find other potential mechanism responsible for IL20RB's high expression in ccRCC. Methylation seems a promising one. As is shown in Figure 7(a), there is a strong negative correlation between IL20RB expression and IL20RB methylation. The Pearson correlation coefficient is -0.83, with a negligible *p* value as small as 1.05*e* − 103, and the Spearman correlation coefficient is -0.72 with a *p* value of 3.86*e* − 66. Then, we explored the correlations between methylation and clinical characteristics; results suggest that the promoter methylation level of IL20RB in tumor tissues is significantly lower than that in normal tissues (Figure 7(b)). When talking about the individual cancer stage and tumor grade, there exists a trend that lower methylation comes with higher tumor stage and tumor grade, which is more obvious in tumor grade (Figures 7(c) and 7(d)).

**Figure 8:**
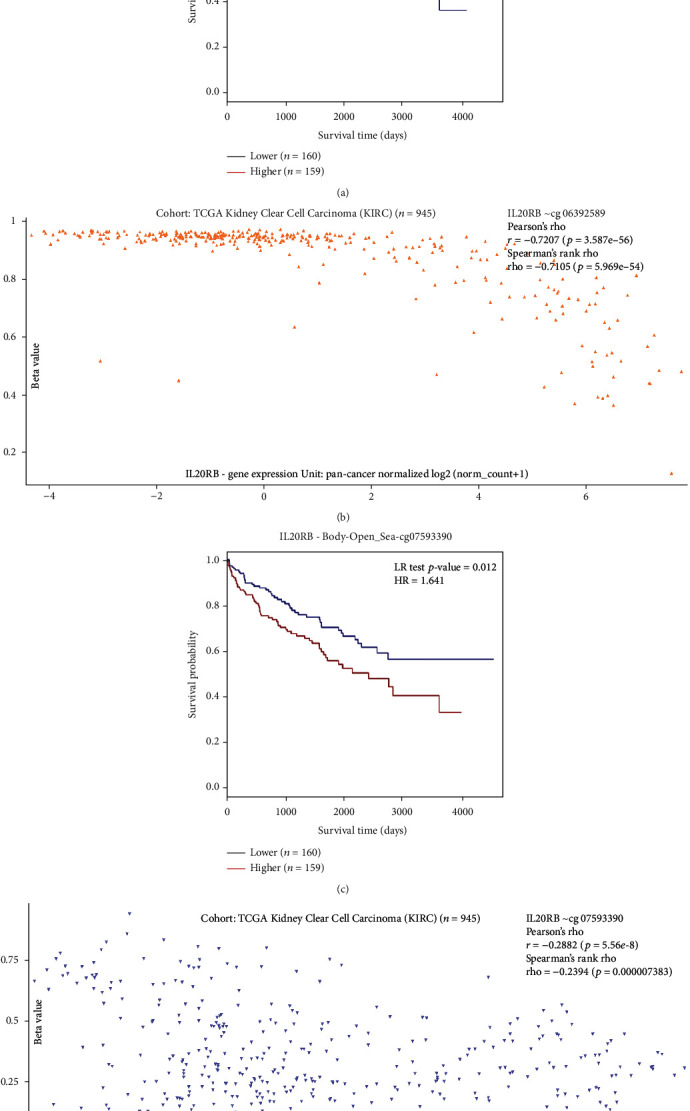
Analysis of each methylation site of IL20RB in MethSurv. (a) cg06392589 shows a significant value of prognosis. (b) Correlation between IL20RB expression and methylation level of cg06392589, analysed by Xena. (c) cg07593390 also shows significant value in prognosis. (d) Correlation between IL20RB expression and methylation level of cg07593390, analysed by Xena.

Further, we aimed to determine the exact methylation site that is potentially responsible for IL20RB's upregulation, which may give insight for therapeutic research. An online tool named MethSurv was used to explore the correlations between survival and methylation level of each methylation site. There are 4 sites with information available in TCGA-KIRC cohort. They are cg01910938, cg06392589, cg07593390, and cg 22746584 in Open Sea island. By estimating the correlation with prognosis choosing median as splitting option, cg06392589, whose genomic region is the 1st exon or 5′UTR, was found to be strongly related with prognosis. The *p* value of cg06392589 is 4.2*e* − 05 and the hazard rate (HR) is 0.441 which means the patients in the high methylation group have a better survival (Figure 8(a)). And there is a strong negative correlation between IL20RB expression and methylation level of cg06392589 as expected (Figure 8(b)). Apart from cg06392589, only cg07593390 in the body region with a *p* value of 0.012 was found to be related with prognosis. On the contrary, its HR is 1.641 which means the patients in the high methylation group have a worse survival (Figure 8(c)). There exists negative correlation between IL20RB expression and methylation level of cg07593390 which is not as strong as cg06392589 (Figure 8(d)). Considering the fact that DNA hypomethylation is usually the mechanism responsible for upregulation of tumor driver gene, cg06392589 may be a more valuable site as a potential therapeutic target which deserves more attention and intensive research.

### 3.5. Functional Analysis of IL20RB in ccRCC

**Figure 9:**
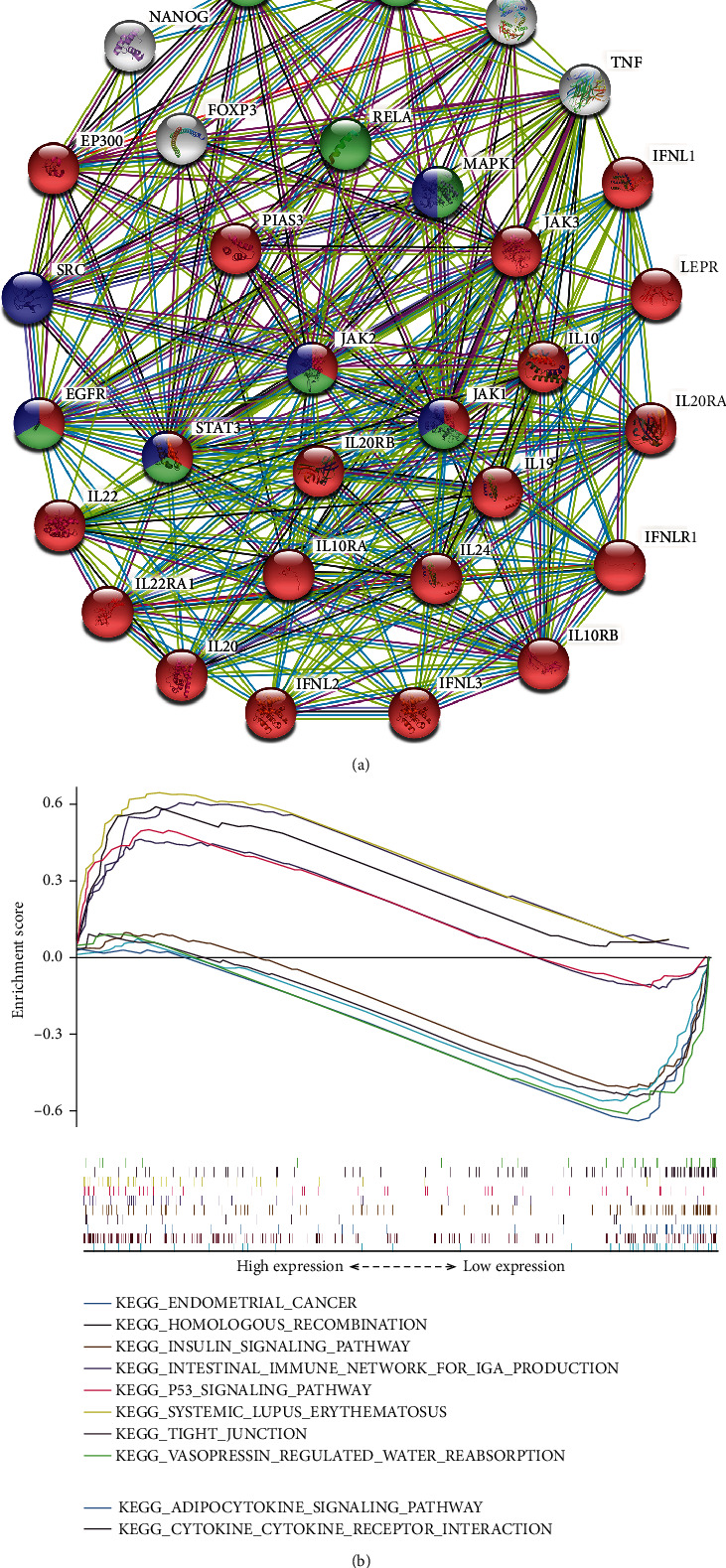
Functional analysis of IL20RB in ccRCC. (a) The PPI (protein-protein interaction) networks of IL20RB are displayed by STRING. Genes involved in the JAK-STAT signaling pathway are in red, genes involved in the PD-L1 expression and PD-1 checkpoint pathway in cancer are in green, and genes involved in EGFR tyrosine kinase inhibitor resistance are in blue. (b) Gene set enrichment analysis between the high and low IL20RB expression groups.

Methylation was successfully identified as a potential mechanism accounting for the upregulation of IL20RB, but there are still many mysteries of IL20RB waiting to be uncovered; the most appealing one with great significance is how IL20RB functions in ccRCC. We firstly constructed the protein-protein interaction (PPI) networks of IL20RB. The network displayed by STRING suggests that IL20RB have a close interaction with members of interleukins, including IL19, IL20, and IL24; with components of interleukin receptors, including IL20RA and IL22RA1; and with members involved in the JAK-STAT signal pathway, including JAK1, JAK2, JAK3, and STAT3. These indicate that IL20RB may mainly function as interleukin receptor and promote cancer development by the JAK-STAT pathway. In addition, KEGG pathways analysis suggests that this network is enriched in pathways including the JAK-STAT signaling pathway, PD-L1 expression and PD-1 checkpoint pathway in cancer, and EGFR tyrosine kinase inhibitor resistance (Figure 9(a)). These indicate that IL20RB may play a role in the emerging immunotherapy and may be a potential indicator to predict the effect of the currently used tyrosine kinase inhibitor therapy.

To further investigate the difference between IL20RB high and low expression group, we conducted GSEA to identify the pathways enriched in two groups, respectively. As is shown in Figure 9(b), the five most functional enrichment pathways of KEGG in the high expression group are cytokine-cytokine receptor interaction, systemic lupus erythematosus, intestinal immune network for IgA production, p53 signaling pathway, and homologous recombination. These pathways are mainly involved in immune response which may mean the tumor tissues are worse differentiated and have a higher malignancy to induce a relatively more violent immune response. On the contrary, the following pathways were mostly enriched in the low expression group: tight junction, insulin signaling pathway, adipocytokine signaling pathway, endometrial cancer, and vasopressin-regulated water reabsorption. These pathways are more likely to function in normal renal cells which may indicate that tumor samples of the IL20RB low expression group are more like normal tissues and have a better histologic differentiation thereby a lower malignancy phenotype. These results enhance our confidence to consider IL20RB as a tumor driver gene in ccRCC.

### 3.6. Immune Infiltration Analysis of IL20RB in ccRCC

**Figure 10:**
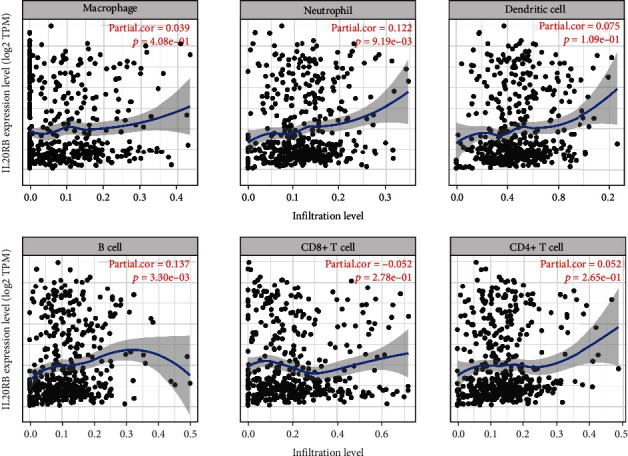
Immune infiltration analysis of IL20RB in ccRCC. Correlations between IL20RB expression and six immune cell types in the tumor microenvironment analysed in TIMER.

With the help of TIMER, we were able to find out the correlations between IL20RB expression and six immune cell types in the tumor microenvironment. Among B cell, CD4 T cell, CD8 T cell, neutrophil, macrophage, and dendritic cell, only B cell and neutrophil were estimated to have significant relationships with IL20RB expression, and both of them are positive correlation which means high expression of IL20RB is supposed to come up with more B cells and neutrophils infiltrating in the tumor microenvironment (Figure 10).

**Figure 11:**
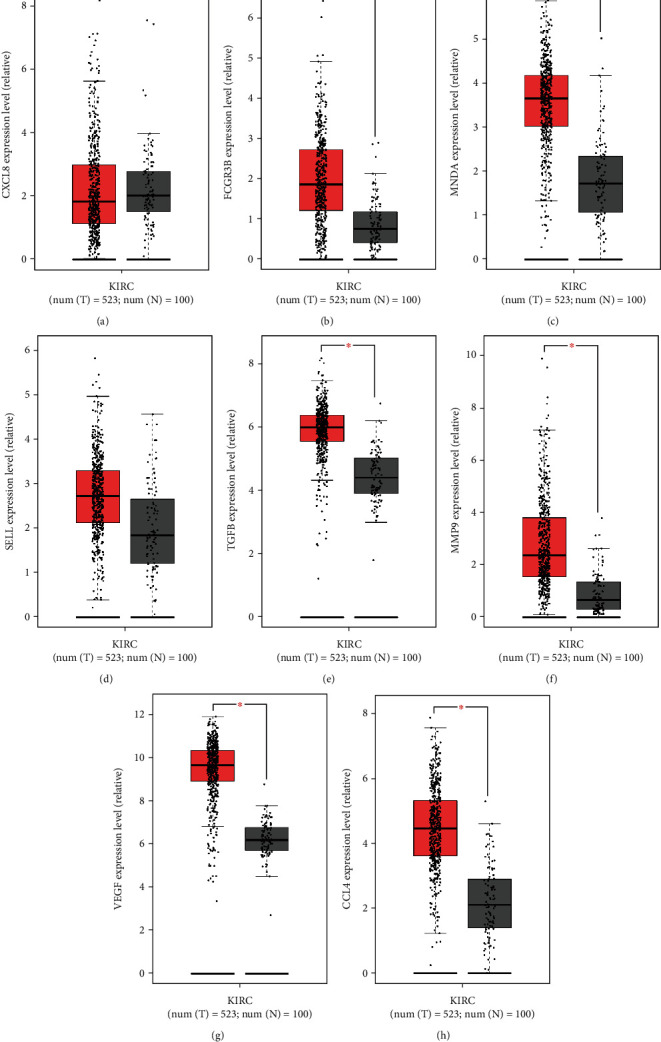
Expression of neutrophil-related genes in ccRCC. Expression of 4 cell markers of neutrophils in renal cell carcinoma: they are (a) CXCL8, (b) FCGR3B, (c) MNDA, and (d) SELL, tumor in red and normal in grey (match TCGA normal and GTEx data). Expression of function-related molecules of tumor-associated neutrophils: there are (e) TGFB, (f) MMP9, (g) VEGF, and (h) CCL4.

As there is growing body of evidence that implies tumor-associated neutrophils (TAN) could play a vital role in many tumor types, we inferred that neutrophils could have something to do with ccRCC. From the CellMarker website [45], we got 4 cell markers of neutrophils in renal cell carcinoma, and by GEPIA, we found FCGR3B and MNDA are significantly highly expressed in ccRCC tumor samples; SELL is also relatively highly expressed though without statistical significance (Figure 11(a)). Additionally, we explored the potential function-related molecules of TAN in GEPIA. Results support that TGF-*β*, MMP9, VEGF, and CCL4 are all significantly highly expressed in tumor samples [46] (Figure 11(b)). These results increased the possibilities that IL20RB may interact with TAN in the progression of ccRCC.

**Figure 12:**
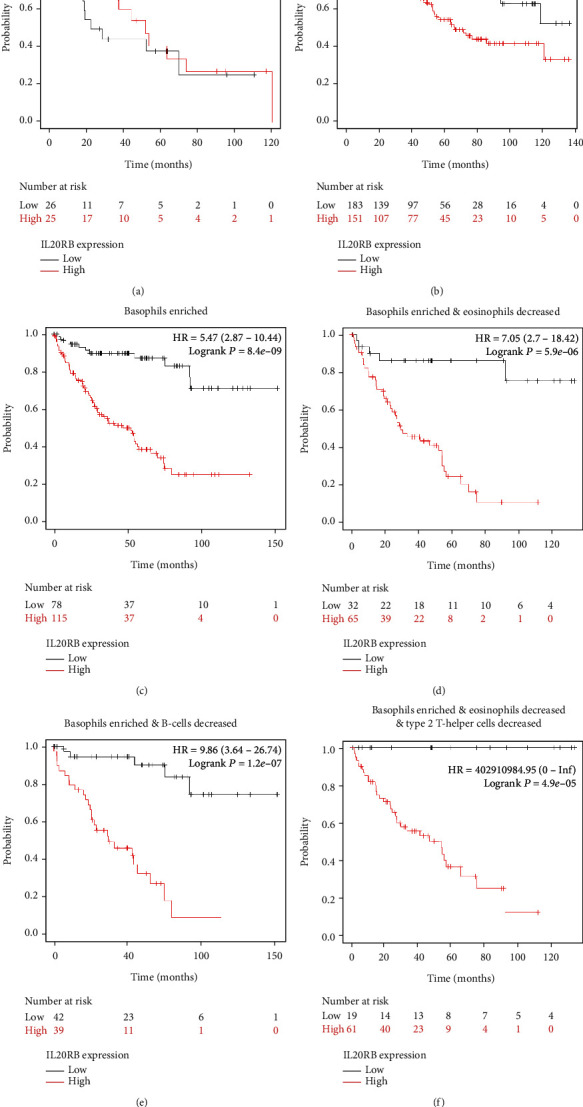
Prognosis value of IL20RB in patients of different immune microenvironments. Prognosis value of IL20RB in patients with (a) type 2 T helper cells enriched, (b) basophils decreased, (c) basophils enriched, (d) basophils enriched and eosinophils decreased, (e) basophils enriched and B cells decreased, and (f) basophils enriched and eosinophils decreased and type 2 T helper cells decreased.

At the same time, we further estimated the prognostic role of IL20RB in different immune microenvironments. Results show that the prognosis predicting ability of IL20RB proves intimately related with tumor immune microenvironment. In most conditions, highly expressed IL20RB predicts a worse survival, but in a minority of circumstances such as type 2 T helper cell-enriched samples, there is no statistical significance between the high and low IL20RB expression groups (Figure 12(a)). In patients with basophils decreased, the HR of the IL20RB high expression group to the low expression group is 2.42, log-rank *p* is 2.2*e* − 05 (Figure 12(b)); on the other hand, in patients with basophils enriched, the HR increases to 5.47 with log-rank *p* as small as 8.4*e* − 09 (Figure 12(c)). This indicates that IL20RB can do better in patients with a basophil-enriched microenvironment. Additionally, we explored more specific conditions and found that, in basophil-enriched but eosinophil-decreased samples, the HR of the IL20RB high expression group to the low expression group is 7.05 (Figure 12(d)); and in basophil-enriched but B cell-decreased samples, the HR of the IL20RB high expression group to the low expression group is 9.86 (Figure 12(e)); in a very specific condition with basophils enriched, eosinophils decreased, and type 2 T helper cells decreased, the HR is as big as 402910984.95, and the log-rank *p* is as small as 4.9*e* − 05, though the patients involved are not in a large number (Figure 12(f)). Primarily, we can conclude that the prognosis predicting ability of IL20RB is closely related with the tumor immune microenvironment in ccRCC.

## 4. Discussion

Clear cell renal cell carcinoma is a type of severe malignant tumor threatening the health of human beings and has drawn lots of attention from many researchers. Nowadays, our efforts have largely improved the management of ccRCC patients. Apart from surgical resection, molecular targeted drugs, including drugs targeting VEGF, PDGFR, EGFR, and mTOR, are available for patients with high recurrence risk and for patients lacking opportunity for radical resection [47, 48]. However, the therapeutic effects are still not very satisfying and there is no doubt that ccRCC is a heterogeneous disease, which means more researches are needed to find novel targets for better diagnosis and therapy.

In this study, we are mainly focused on IL20RB, whose role in ccRCC is still unknown but has shown its potential as a novel biomarker in some human cancers. Firstly, we confirmed that IL20RB is unquestionably upregulated in ccRCC using three independent databases, including TCGA, GEO, and Oncomine. Then, we verified the prognosis relationship in TCGA cohorts, which suggests high expression predicts worse survival. Further clinical correlation analysis supports that IL20RB have positive relationship with characteristics that can reflect recurrence risk such as tumor stage and tumor grade. These results imply that IL20RB may act as a novel tumor driver gene in ccRCC.

Then, we determined to find out the underlying mechanisms responsible for IL20RB's high expression in ccRCC. As genetic alternation rate is extremely as low as 0.9% and there lacks survival correlation between altered and unaltered groups, we turned to methylation. Fortunately, methylation analysis supports that a low methylation level of IL20RB is strongly related with high IL20RB expression. Additionally, cg06392589 was identified as an important methylation site which is a powerfully potential target for the development of new therapy.

To uncover the biological processes IL20RB may be involved, functional analysis was conducted by STRING and GSEA. Results indicate that IL20RB is more likely to act as a cytokine receptor, and tumor samples with high IL20RB expression are more likely to enrich pathways related to immune response such as cytokine-cytokine receptor interaction and intestinal immune network for IgA production. Extensive studies have revealed the relationship between tumor development and immune system [49]. We further investigated the immune infiltration of ccRCC, and we found that IL20RB expression is positively related with B cells and neutrophils. On the one hand, the function of B cells in the tumor microenvironment is not fully studied at present, even though there are some studies indicating B cell could promote cancer metastasis [50]. On the other hand, tumor-associated neutrophils have been studied a lot and many mechanisms have been uncovered to explain the function of TAN [51]. Therefore, in our study, we mainly focused on neutrophils and found the cell markers of neutrophils in renal cell carcinoma are highly expressed. TGF-*β* was reported to be necessary for neutrophils to promote tumor development [52], and our study verified the high expression of TGF-*β* in ccRCC. MMP9 [53], VEGF [54], and CCL4 [55] were found to be employed by neutrophils to promote tumor development, and our study verified them to be upregulated in ccRCC. These results indicate that IL20RB may work with neutrophils to promote the development of ccRCC. Thus, new combination therapies targeting both IL20RB and neutrophils may be promising. Furthermore, our analysis reveals that the predicting ability of IL20RB is related with the immune microenvironment. We found some specific immune microenvironment in which blocking IL20RB may bring more benefits, such as a microenvironment with basophils enriched, eosinophils decreased, and type 2 T helper cells decreased.

There are some limitations in our study as well. Firstly, the analyses were mainly made based on TCGA-KIRC cohort; some results lack validation in external independent cohorts of ccRCC. Secondly, the analyses were mainly made by bioinformatic methods; further in vitro as well as in vivo studies are needed to support these findings. In addition, our study is an early and primary exploration of the IL20RB and there is still a long way to translate this academic research into clinical practice.

As far as we know, many genes have been proposed as biomarker in ccRCC. For example, Zhang and his colleague have discovered several interested genes and demonstrated their potential as target for ccRCC. NEK2, NIMA-related kinase 2, was found to be overexpressed in ccRCC and high expression of NEK2 was associated with a poor prognosis [56]. CD146 was identified to be elevated in ccRCC tissues, and high CD146 expression was associated with poor prognosis in patients with ccRCC [57]. FGL1, fibrinogen-like protein 1, which is known as a novel potential immune checkpoint target [58], was recognized to be upregulated in tumor tissues and plasma specimens of ccRCC patients, and high FGL1 expression predicted a poor prognosis. In fact, there are many genes that have been identified as potential biomarkers in ccRCC, such as ZNF433 [59], AQP9 [60], SPINK13 [61], and DEF6 [62]. These works are different from each other for their interested and focused genes vary, but all genes identified are treasures for they have enhanced our understanding of ccRCC. In this study, we mainly focused on IL20RB's role in ccRCC. We are pleased to add our own efforts to uncover a little secret of the complex and extensive changes happened in the origin and progression of ccRCC. Anyway, our discovery is relatively insightful as well as inspiring and we will feel gratified if the small steps of our exploration do benefit patients in the future.

## 5. Conclusions

IL20RB is dysregulated in many human tumors and extremely upregulated in ccRCC. Methylation could be responsible for the upregulation, and cg06392589 methylation site could be the main target. Both high expression and low methylation of IL20RB predict worse survival in ccRCC patients, and both have a strong positive correlation with clinical features. IL20RB may play as a tumor driver gene in ccRCC by participating immune response; there seemingly exists crosstalk between IL20RB and neutrophils. Additionally, the immune microenvironment could influence the prognosis predicting ability of IL20RB. Above all, IL20RB is identified as a novel prognostic and therapeutic biomarker in clear cell renal cell carcinoma from a bioinformatic perspective.

## Data Availability

The results published or shown here are in whole or part based upon data generated by TCGA Research Network: https://www.cancer.gov/tcga. The results published or shown here are in whole or part based upon data generated by Expression Atlas: https://www.ebi.ac.uk/gxa. The results published or shown here are in whole or part based upon data generated by the Cancer Cell Line Encyclopedia: https://sites.broadinstitute.org/ccle.
